# Polymorphisms in the Human Cytochrome P450 and Arylamine *N*-Acetyltransferase: Susceptibility to Head and Neck Cancers

**DOI:** 10.1155/2013/582768

**Published:** 2013-09-16

**Authors:** Rim Khlifi, Olfa Messaoud, Ahmed Rebai, Amel Hamza-Chaffai

**Affiliations:** ^1^Research Unit on Toxicology and Environment, Sfax University, 3018 Sfax, Tunisia; ^2^Bioinformatics Unit, Centre of Biotechnology of Sfax, Sfax University, 3018 Sfax, Tunisia; ^3^Biomedical Genomics and Oncogenetics Laboratory LR11IPT05, University of Tunis El Manar, 1002 Tunis, Tunisia

## Abstract

The occurrence of head and neck cancer (HNC) is associated with smoking and alcohol drinking. Tobacco smoking exposes smokers to a series of carcinogenic chemicals. Cytochrome P450 enzymes (*CYP450s*), such as *CYP1A1*, *CYP1B1*, and *CYP2D6*, usually metabolize carcinogens to their inactive derivatives, but they occasionally convert the chemicals to more potent carcinogens. In addition, via *CYP450* (*CYP2E1*) oxidase, alcohol is metabolized to acetaldehyde, a highly toxic compound, which plays an important role in carcinogenesis. Furthermore, two *N*-acetyltransferase isozymes (*NATs*), *NAT1* and *NAT2*, are polymorphic and catalyze both *N*-acetylation and *O*-acetylation of aromatic and heterocyclic amine carcinogens. Genetic polymorphisms are associated with a number of enzymes involved in the metabolism of carcinogens important in the induction of HNC. It has been suggested that such polymorphisms may be linked to cancer susceptibility. In this paper, we select four cytochrome *P450* enzymes (*CYP1A1*, *CYP1BA1*, *CYP2D6*, and *CYP2E1*), and two *N*-acetyltransferase isozymes (*NAT1* and *NAT2*) in order to summarize and analyze findings from the literature related to HNC risk by focusing on (i) the interaction between these genes and the environment, (ii) the impact of genetic defect on protein activity and/or expression, and (iii) the eventual involvement of race in such associations.

## 1. Introduction

Head and neck squamous cell carcinoma (HNSCC) is the fifth most common cancer worldwide and is associated with low survival and high morbidity when diagnosed in advanced stage [1, 2]. This type of cancer accounts for almost 500,000 newly diagnosed cancer cases per year [3, 4]. Epidemiological studies have shown that HNSCC occurs through a complex multistage process that may involve exposure to a combination of carcinogens from cigarette smoking [5, 6], alcohol consumption [7], or tobacco chewing [8, 9]. As in some regions of the world, these toxic agents are responsible for about 75% of all cancer cases. HNSCC is used to be considered as a tobacco-induced and a preventable cancer [4, 10]. The hypothesis that genetic susceptibility or predisposition is of important role in head and neck cancer (HNC) etiology is highly supported by case-control studies of several phenotypic and genotypic assays [11–13]. Some studies stated that gene-environment interactions in relation to HNSCC are linked to genes involved in metabolism enzymes for alcohol and tobacco smoke constituents [14]. Polymorphisms in the genes encoding these enzymes, by altering their expression and function, may increase or decrease carcinogen activation/detoxification, followed by modulation of cancer risk [15, 16].

Polymorphisms in the carcinogen-metabolizing genes have been analyzed on individual basis [17]. Several studies have addressed the relationship between the genetic polymorphisms of enzymes involved in the metabolic activation of carcinogens and the occurrence of HNSCC [15, 18, 19]. Genetic polymorphisms in cytochrome P450 (i.e., *CYP1A1, CYP1B1, CYP2D6, *and *CYP2E1*) and *N-*acetyltransferase (*NAT1* and *NAT2*) enzymes involved in the biotransformation of the carcinogenic constituents of tobacco have been shown to be the risk factors involved in HNSCC [20–23]. These enzymes are very important with respect to the metabolism of a large number of xenobiotic carcinogens (Table 1). Carcinogens present in tobacco smoke such as polycyclic hydrocarbons including the prototype of this chemical class, benzo(a)pyrene [24, 25], and tobacco-specific nitrosamines (TSNAs) have been implicated in HNC etiology in smokers [26]. It was previously suggested that acetaldehyde, the first metabolite of alcohol when orally ingested, is involved in alcohol-related cancer induction. Nevertheless, carcinogenic pathway of alcohol is not elucidated [27].

In the present paper, we summarize results of studies (published up to February, 2013) dealing with the association between the genetic variations in genes coding for phases I and II carcinogen metabolism enzymes (*CYP1A1, CYP1BA1, CYP2D6*, *CYP2E1*, *NAT1, *and *NAT2*) and the increased risk of head and neck cancer development.

## 2. Cytochrome P450 (*CYP450*)

The determinant factors for HNSCC development remain unclear. Although the importance of tobacco and alcohol consumption as risk factors suggests that genes encoding detoxifying enzymes are susceptibility candidates, several data have not confirmed associations between these enzymes and the occurrence of HNSCC. Previous studies suggest that various CYP genotypes are linked with its outcome rather than its susceptibility [28, 29].

### 2.1. *CYP1A1*

The human enzyme *CYP1A1 *is the most active among the CYPs in metabolizing procarcinogens, particularly, the polycyclic aromatic hydrocarbons (PAHs), into highly reactive intermediates [30]. When these compounds bind to DNA and form adducts, they may contribute to carcinogenesis. Despite the fact that PAHs are ubiquitous in the environment, remarkable sources of exposure such as smoking, certain occupations, and air pollution may lead to the greatest concern [31]. The aromatic hydrocarbon receptor is a key activator of the *CYP1A1* gene [32, 33]. PAHs were classified among important toxicants as they induce *CYP1A1* gene and act as precarcinogenic substrates [34, 35]. The relationship between *CYP1A1* variants and cancer risk has been investigated in several studies [18]. *CYP1* enzymes are coupled to phase II detoxification *in vivo*. It has been proposed that, compared with other *CYP1* enzymes, *CYP1A1* is more tightly coupled to phase II metabolism and plays a more important role *in vivo* in detoxification than toxin activation [36].

A recent study confirmed the importance of tobacco smoking as the main risk factor for the upper aerodigestive tract (UADT), indicating that about 68% of cancers can be attributed to this risk factor. A significant association between metabolizing phase I genes (*CYP1A1*) and UADT cancers was found [37]. Nagaraj et al. [38] identified molecular factors which contribute to the increased risk of smokers for oral squamous cell carcinoma (OSCC). In fact, they evaluate gene expression profile change according to cigarette smoke condensate in normal epidermal keratinocytes, oral dysplasia cell lines Leuk1 and Leuk2, and a primary oral carcinoma cell line 101A. Their results have shown that treatment by cigarette smoke condensate acts on several cell types and usually leads to overexpression of *CYP1A1*. These findings support the hypothesis that cigarette smoke condensate is widely involved in the activation of procarcinogens. These results are similar to those of Chi et al. [39] and those of Wen and Walle [40]. 

A functional role has been previously assigned to two nonsynonymous polymorphisms in the *CYP1A1* gene. The first one is an adenine (A) to guanine (G) substitution at codon 462 in exon 7 (Ile462Val, rs1048943). The second one is a thymine (T) to cytosine (C) transition (rs4646903) [41]. This last mutation changes a restriction site for the *MspI* enzyme, thus resulting in three genotypes: a predominant homozygous allele (genotype A, TT), a heterozygous allele (genotype B, TC), and a homozygous rare allele (genotype C, CC) [42]. Contrary to genotype C, genotype A abolishes the restriction enzyme site of *MspI*. The exon 7 restriction-site polymorphism resulted in three genotypes: the predominant homozygous (Ile/Ile), the heterozygous (Ile/Val), and the rare homozygous (Val/Val). Another mutation, *CYP1A1 *T6235C (m1), located in the 3′ end of this gene, is considered as a polymorphism for the restriction endonuclease *MspI* and results in a mutant *CYP1A1* allele designated as CYP1A1*2A. Three additional polymorphisms have also been reported in exon 7 of the *CYP1A1* gene. The first one is a *CYP1A1* 4889AG (m2) transversion responsible for the replacement of Ile by Val at position 462 in the mutant form of the protein, and it is known as CYP1A1*2B. The second polymorphism is caused by a *CYP1A1 *T5996C (m3) transition in the 3′ noncoding region of the gene which is known as CYP1A1*3. The last one is located at position 4887 and is a transversion, a *CYP1A1* 4887C/A (m4), that results in a mutation of Thr to Asn at codon 461 (CYP1A1*4) [41, 43, 44]. Among these four polymorphisms, the *MspI* at the 3′ flanking region has been reported in many epidemiological studies to be associated with cigarette smoking-related cancer risk in some but not all studies [18, 41, 43, 45–52].

**Figure 1:**
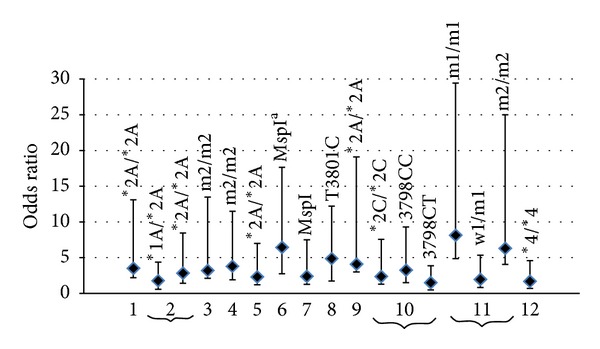
Odds ratios (OR) for HNC obtained from 12 *CYP1A1* studies. Bars indicate 95% confidence intervals (CI), while individual SNPs in each study are labeled for each vertical line, and study numbers are indicated at the bottom (1: [69]; 2: [66]; 3: [64]; 4: [63]; 5: [62]; 6: [65]; 7: [68]; 8: [89]; 9: [55]; 10: [60]; 11: [79]; 12: [56]). ^a^Smokers.

 Several studies have been since performed examining the potential association between the polymorphic *CYP1A1 *(*MspI* and/or exon 7) and the HNC occurrence (Figure 1). In the Brazilian patients, a tendency of increased oral cancer risk among *CYP1A1* genotypes (426Val/Val) that compared both with the wild-type homozygous (OR = 2.85) and heterozygous (OR = 2.61) ones was found by Marques et al. [53]. The *CYP1A1* (426Val/Val) genotype was found three times more frequent than in controls in 3% of oral cancer patients. In spite of the absence of any statistical significance, these results strongly supported the previous ones showing that the mutant allele *CYP1A1* 426Val is related to an increased risk of oral cancer in Caucasians, in the United States [47], among Asian populations [54], and in Indians [19]. It was also reported that the *CYP1A1* 4889 A/G genotype [Ile462Val (rs1048943)] is more frequent in the group of white HNSCC patients (10%, *n* = 108) than in white controls (7%, *n* = 165) [15]. However, for genotype heterozygous, the moderate increase in the HNSCC risk was not statistically significant. Nevertheless, it was reported an overrepresentation of the *CYP1A1* 4889G allele among the nonsmoking Caucasian patients with oral cancer [47] and among the Japanese HNSCC patients [55]. For the Polish patients, the increased frequency of the CYP1A1*4 allele and the CYP1A1*4/*4 genotype (*CYP1A1* Thr461Asn) supports its association with HNC and might be specific for laryngeal SCC [56]. However, Reszka et al. [57] and Amtha et al. [58] suggested no significant increase in HNC risk in the Polish and Indonesian patients, respectively, with the *CYP1A1 *462Val alleles (OR = 1.60 and 0.70, resp.). Moreover, in a recent meta-analysis study, no association between Ile462Val polymorphism and HNC risk was found [59].

 Polymorphisms located in the *CYP* gene result in the enzyme activity increase [60]. The homozygous *CYP1A1* (*MspI*) mutations are present in 7% to 10% of the white population and in up to 33% of the Japanese population. These homozygous (m2/m2) polymorphisms were associated with a high susceptibility to SCC of the lung or UADT according to some researches [61, 62]. To investigate the association between *CYP1A1 *polymorphism *(MspI) *and risk for OSCC in the Korean [63] and the Indian [64] populations, many studies have been conducted, and they found that the risk for oral cancer was significantly increased in subjects of these populations with the homozygous *CYP1A1* (m2/m2) genotype (Indian: OR = 3.2, 95% CI = 1.10–10.28, and *P* = 0.05; Korean: OR = 3.8, 95% CI = 1.9–7.7, and *P* = 0.023), regardless of smoking history (smokers: OR = 4.4, and 95% CI = 1.2–16.3; nonsmokers: OR = 4.9, and 95% CI = 1.9–12.5). Recently, in Liu et al. [59] meta-analysis, a significant association between *MspI *SNP and HNC risk was found (95% CI = 1.15–1.57; *P* < 0.001). This effect was found to be more pronounced in smokers (OR = 2.98, 95% CI = 1.69–5.26, and  *P* < 0.001), thus demonstrating that gene-smoking interaction that intensifies carcinogenesis might exist [59]. Additionally, Sam et al. [65], found that the individuals polymorphic for *CYP1A1 MspI* revealed an increased risk for UADT cancers than that ascribed to a single susceptible gene among tobacco users in the Indian population (OR = 6.43; 95% CI = 3.69–11.21). Moreover, in a previous study, Sam et al. [66] found that CYP1A1*1A/*2A and *2A/*2A polymorphic genotypes are associated with an enhanced risk to UADT cancers, in particular, among the habitual tobacco smokers and chewers carrying mutant genotypes in the Indian population (OR = 1.76; 95%  CI = 1.19–2.60 and OR = 2.83; 95% CI = 1.43–5.61, resp.). Furthermore, Olivieri et al., [67] Figaro Gattá et al. [68], Tanimoto et al. [69], and Singh et al. [23] reported that the Brazilian, the Japanese, and the North Indian patients carrying *CYP1A1* (*1A/*2A) genotype presented an increased HNSCC risk. However, no statistically significant difference in the CYP1A1*2A allele and in the CYP1A1*2A/*2A genotype frequency was found in Gajecka et al. study [56].

Many researches focused on the association of *CYP1A1 *polymorphism with susceptibility to laryngeal cancer. Unfortunately, their results were inconsistent and inconclusive. *CYP1A1 MspI *polymorphism was found to be a risk factor for laryngeal cancer in Caucasians (OR = 1.29) but not in Asians (OR = 1.38) [70]. Variant genotypes of *CYP1A1* might not be considered as risk factors for oral cancer [70]. Moreover, Tai et al. [71] studied *CYP1A1* polymorphisms in the Chinese patients with laryngeal and hypopharyngeal SCC and control subjects. They found an increased risk associated with the *CYP1A1 *3798CC genotype (OR = 2.39; 95% CI = 1.11–5.16), compared with the TT genotype [71]. In other investigations, no such association was found [72–74]. In the Gronau et al. report [75], a German case-control study, the authors found that the homozygous mutation and the *MspI* restriction site in exon 7 are present only once in the control group and that no patient revealed this genotype. Furthermore, the genotype frequencies at the *CYP1A1* gene loci investigated in other German case-control studies showed no differences between these groups, suggesting a lack of influence of these genes in the susceptibility to laryngeal cancer [28, 76]. The association of nasopharyngeal cancer (NPC) in Taiwan with *CYP1A1 MspI* genetic polymorphism was studied [77], and no significant associations of the examined genotypes with NPC risk were noted. Moreover, a recent study [78] of two SNPs in *CYP1A1 *m1*   * [*MspI *(rs4646903)] and *CYP1A1 *m2*   * [Ile462Val (rs1048943)] in a total of 457 Cantonese nuclear families, consisting of 2134 members, has concluded that there is no absence of any statistical significance between m1 polymorphism and susceptibility to NPC. However, m2 polymorphism might be associated with NPC in the Cantonese nuclear families (*P* = 0.045) [78].

It is noteworthy that all studies on the relationship between *CYP1A1* genotype and cancer have focused on each polymorphism separately. Having global information regarding the individual haplotype could give better clarification of such associations. Recently, Sabitha et al. [79] examined for the first time the association of three SNPs in the *CYP1A1 MspI* locus (m1/m1, w1/w1, and m2/m2) with HNC risk. They found that individuals carrying at least one *CYP1A1 *m1*   * or m2 variant allele were at a 2-fold elevated risk for HNC and concluded that *CYP1A1* is an important determinant in susceptibility to tobacco-induced HNC among Indians [79]. Cigarette smoke has been shown to upregulate *CYP1A1* under *in vitro* conditions as well as in smokers [38, 39, 80]. In five earlier different studies investigating *CYP1A1 *genotype-smoking interactions [48, 81–84], two have reported evidence of an interaction [81, 84]. Further few studies [28, 72, 85, 86] did not find a relationship between pack-years of smoking and risk of HNSCC among cases with the *CYP1A1 MspI* polymorphism. But recently, Sabitha et al. [79] found association between pack-years of smoking and risk of HNSCC among cases with the *CYP1A1 MspI* polymorphism. Heavy smokers showed an increased risk for HNC in association with both m1 and m2 mutations. The OR of HNC for the variant *CYP1A1 *m1*   * genotype, the tobacco smoking, and both factors combined were OR = 4.93, 95% CI = 1.83–13.68; 1.07, 95% CI = 0.16–7.34; 0.60, 95% CI = 0.30–1.18, respectively. Sabitha et al. [79] findings support that *CYP1A1 *m1*   * and *CYP1A1 *m2*   * polymorphisms were associated with smoking-related HNC in India.

Association of more than one SNP in one individual may additively or synergistically contribute to the increased cancer risk. Furthermore, the impact of xenobiotic-metabolizing enzymes and transporters could determine the functional results in the risk of HNC over the independent effects of each single susceptibility gene. It is becoming clearly evident that single gene or single environmental factor cannot explain susceptibility to diseases with complex etiology such as HNC. Expression of these enzymes might be one of the reasons for interindividual differences in HNC risks. In a recent study, Masood et al. [87] studied the expression of *CYP1A1 MspI* in HNC tumor and normal healthy tissues, and the relationship with stages of HNC in the Pakistani population. They found that the* CYP1A1 *mRNA is less expressed in head and neck carcinoma compared with adjacent normal tissue (OR = 4.5, 95% CI = 1.5–13.4). *CYP1A1* expression was downregulated according to tissue stage as follows: 62.5% in tissues of stage 1, 72.7% in tissues of stage 2, 60% in tissues of stage 3, and 100% in tissues of stage 4. Therefore, it is very obvious to conclude that CYP expression is involved in the carcinogenesis by a pathway that is still not elucidated.

Recently, Sharma et al. [88] explored the North Indian population by a multifactor dimensionality reduction method in order to determine potential gene-environment and gene-gene interactions that predispose to HNC. They observed significant gene-gene interactions among *GSTM1 *copy number variants and *CYP1A1* T3801C (rs4646903) variant among smokers. This method showed that the combining three factors, smoking status, *CYP1A1* T3801C, and *GSTM1* copy number variants, conferred more than 4-fold increased risk of HNC (OR = 4.89; 95% CI = 3.15–7.32; *P* < 0.01). Therefore, genetic variants in tobacco-metabolizing genes may contribute to HNC risk through gene-gene and gene-environment interactions. In a previous study of Sharma et al. [89] research group, epigenetic modifications of genes involved in carcinogen metabolism pathway, *CYP1A1, CYP2A13,* and *GSTM1*, were assessed by evaluating the role of aberrant hypermethylation as well as its relation to tobacco and alcohol consumption. In addition, *CYP1A1* and *CYP2A13* polymorphisms were also investigated in the Indian population. Results of this study showed that hypermethylation of *CYP1A1* and *GSTM1 *showed significant association with HNC (*P* = 0.027, and *P* = 0.010, resp.). They also showed a significant interaction between smoking and methylation status of *CYP1A1 *and *CYP2A13* in HNC (*P* = 0.029, and *P* = −0.034, resp.). So hypermethylation of carcinogen metabolism pathway genes is associated with an increased risk of HNC regardless of the smoking status [89].

In a recent case-control Indian population study [90], the *CYP1A1* (*2A and *2C), *CYP2E1* (*1B, *5B, and *6), and *GST *(M1, T1, and P1) adenosine triphosphate-binding cassette B1 3435C > T (ABCB1) polymorphisms were studied. Results showed a high risk of gene-gene interactions with the concurrent deletions of *GSTT1 *and *GSTM1* genotypes associated with variant genotypes of CYP1A1*2A (OR = 8.21; 95% CI = 1.91–49.48), *GSTT1* and *GSTM1*-deficient genotypes with CYP2E1*1B variant genotypes (OR = 6.73; 95% CI = 1.32–22.81), and a very high risk with the combined variant genotypes of CYP1A1*2A, *GSTT1,* and *ABCB1* (OR = 11.14; 95% CI = 2.70–46.02). Thus, showing that interaction with many drug-metabolizing enzymes and transporter proteins is of a high risk for UADT cancers compared with that of a single susceptible gene [90]. The interaction between phase II deficient enzymes and a phase I hyperactive enzyme (*CYP1A1*) is of interest as it can lead to a larger amount of toxic compounds that may play a crucial role in the initiation or progression of UADT cancers. The risk of cancers is frequently higher in individuals with combined mutant genotypes of CYP1A1*2A and *GSTM1 *null genotype than in those with *CYP1A1* or *GSTM1* gene alone. The interaction between *CYP1A1* and *GSTM1* is so important. In fact, it can be related to *CYP1A1* induction [91]. The significant risk for oral cancer among carriers of both CYP1A1*2A homozygous variant and *GSTM1* null genotype previously suggested by Anantharaman et al. [64] was also supported by Indian, Japanese, Korean, and Brazilian studies [62, 63, 68, 69].

### 2.2. *CYP1B1*

Human *CYP1B1 *is located on chromosome 2 at the 2p21-22 region [92, 93]. The length of its genomic DNA is 12 kilobases (kbs), and the length of its mRNA is *≈*5.2 kb. The *CYP1B1 *enzyme (cytochrome P450, family 1, subfamily B, and polypeptide 1) is a hemethiolate monooxygenase involved in metabolizing xenobiotics, such as polycyclic aromatic hydrocarbons (PAHs) [92]. At transcriptional level, *CYP1B1* gene is activated by PAHs that constitute the major constituents of cigarette smoke and tobacco, hence making it responsive to smoked and smokeless (chewing) tobacco [40, 92, 94]. As *CYP1B1* is crucially involved in the bioactivation of chemically diverse tobacco-related procarcinogens to reactive metabolites, its expression is considered as a significant parameter of carcinogenesis [95]. Other expression studies showed that *CYP1B1* is overexpressed in several human tumors in comparison with normal tissues [94, 96, 97]. It was also demonstrated the implication of many allelic variations in *CYP1B1* in modulating the incidence of several types of cancers [98, 99]. Therefore, *CYP1B1* played an important role in carcinogenesis.

In humans, *CYP1B1* locus has been demonstrated to be genetically polymorphic where many mutations have been identified in *CYP1B1* gene so far [100]. Four nonsynonymous single-nucleotide polymorphisms (SNPs) have been described: (i) Arg to Ser at codon 48 (CYP1B1*2) (rs10012), (ii) Ala to Ser at codon 119 (CYP1B1*2), (iii) Leu to Val at codon 432 (CYP1B1*3) (rs1056836), and (iv) Asn to Ser at codon 453 (CYP1B1*4) (rs1800440) [101]. The association of SNPs in *CYP1B1 *with the increased risk of ovarian, endometrial, renal, and prostate cancers as well as smoking-related lung cancer has been reported in the Caucasian and the Japanese populations [102]. Contradictorily, Aklillu et al. [101] have shown that *CYP1B1* variant enzymes differ in their catalytic activity according to the metabolism of 17*β*-estradiol. It has been reported that proteins presenting one of the four common SNPs (Arg48Ser, Ala119Ser, Leu432Val, and Asn453Ser) had slight effects on benzo[a]pyrene-7,8-diol metabolism [103, 104]. This genotype is then considered as a susceptibility factor to develop PAH-induced cancers. Few epidemiological studies aimed at evaluating a possible association between genetic polymorphisms of *CYP1B1 *and susceptibility to HNSCC have been conducted [105, 106].

Two authors have studied the CYP1B1*3 polymorphism and identified the susceptibility factor for HNSCC [21, 26]. In fact, genotype and haplotype frequencies of the four SNPs in *CYP1B1* have been evaluated in HNSCC patients of the Indian population [21]. Singh et al. [21] study indicates a several-fold increase in cancer risk among cases that use tobacco chewing with the variant genotypes of CYP1B1*2 (OR = 8.80; 95% CI = 2.60–29.87; *P* < 0.05) and CYP1B1*3 (OR = 2.74; 95% CI = 1.12–6.70; *P* < 0.05) suggesting that interaction between genes and environment plays an important role in susceptibility to HNSCC. Another significant interaction between the variant genotypes of CYP1B1*2 and cigarette smoking was also found in smoking patients (OR = 2.37; 95% CI = 1.62–4.85; *P* < 0.05). However, for CYP1B1*3 and CYP1B1*4 genotypes (heterozygous and homozygous mutants), no significant interaction regarding smoking with relation to HNSCC has been observed [21]. In contrast to Singh et al. [21], findings, Ko et al. [26] reported the presence of variant genotypes of CYP1B1*3 at a significantly higher frequency in smoking patients compared with healthy smokers, thus suggesting that genotypes of CYP1B1*3 significantly interact with smoking and likely represent a susceptibility factor in smoking related to HNSCC (OR = 4.53; 95% CI = 2.62–7.98; *P* < 0.001). Li et al. [106] failed to find any significant interaction between tobacco smoking and CYP1B1*3 in HNSCC and explained their different results by ethnic backgrounds (Europeans versus American Caucasians). Indeed, there are significant differences in the allele frequency of CYP1B1*2 and CYP1B1*3 variants between Caucasians and Asians [107] and Indians [21]. The variant allele of CYP1B1*2 was more frequent in the Indian controls compared with the Caucasians. This could explain the higher risk for HNSCC in Singh et al. [21] study. However, there were no significant associations between the risk of hypopharyngeal and laryngeal SCC development and *CYP1B1* Leu432Val genotypes [71]. The difference in the genetic background related to the ethnic origin of each population or the involvement of other confounding genetic factors responsible for HNSCC might explain absence of associations.

It is well known that the use of tobacco is often accompanied by alcohol consumption [108]. Many studies have reported a high risk of HNC in alcohol drinkers (adjusted for smoking). Depending on the consumed alcohol amount, this risk varies from less than 2 to 12 folds [9, 109]. Despite the fact that interaction between alcohol and *CYP1B1* genotypes in promoting HNSCC risk is still unknown, it is suggested that tobacco carcinogens are dissolved in alcohol, thus facilitating their access to the mucosa of upper aero-digestive organs [110]. A strong interaction between alcohol consumption and the CYP1B1*2 genotypes for the increased risk to HNSCC was also established [21]. This interaction was associated in patients with a heterozygous genotype of CYP1B1*2 (OR = 6.07; *P* < 0.05) and in patients with the homozygous mutant allele of CYP1B1*2 (OR = 5.24; *P* < 0.05) [21].

Although many polymorphisms of the *CYP1B1* gene have been associated with different cancers, less is known about changes in mRNA expression levels in tumor tissue. The *CYP1B1* gene encodes for a monooxygenase involved in phase I of xenobiotic metabolism. Levels of *CYP1B1* mRNA vary widely from decreased levels in mesothelioma and melanoma to increased levels in prostate and nonsmall cell lung cancer. Hence, *CYP1B1 *enzyme may be an antioncoprotein or an oncoprotein. This depends on what pro-carcinogens are the frequent cancer-causing agents in these tissue types and whether *CYP1B1* serves to activate or inactive them [111–114]. Assessment of *CYP1B1* expression levels in healthy and cancerous tissue types has been well studied. Results showed that *CYP1B1 *is upregulated in numerous cancers such as esophagus, lung, skin, breast, brain, testis, and colon cancers [115]. However, *CYP1B1 *has been detected at low levels in liver, kidney, brain, and eye in healthy adult tissues [92, 95, 116]. In a recent study, Chi et al. [39] evaluated *CYP1B1* mRNA expression in OSCC lines exposed to dibenz[a]pyrene and in healthy oral tissues from smokers and nonsmokers. They noticed that the interindividual variation in inducible *CYP1B1* expression may account in part for variation in tobacco-related OSCC risk. Furthermore, Schwartz et al. [117] found that RNA from brush cytology of hamster oral SCC showed differential *CYP1B1 *expression in dibenz[a]pyrene-induced OSCC. Moreover, Kolokythas et al. [118] demonstrated a downregulation of *CYP1B1 *at the mRNA level only in OSCC from oral brush cytology samples. Similar to Kolokythas et al. [118] findings, Pradhan et al. [119] observed downregulation of *CYP1B1 *in cancerous tissues in comparison with their corresponding healthy tissues as well as in the epithelial dysplasia lesion compared with its matched healthy tissue at the transcriptional level, and in cancerous tissues at the protein level [119]. This difference might be due to different kinds of oral lesions examined by Pradhan et al. [119] and Shatalova et al. [120]. However, an upregulation of *CYP1B1* which included only 19.5% of oral lesions was observed in a recent HNSCC study [120]. These contrasting observations might be due to differences between examined oral lesions by Pradhan et al. [119] and Shatalova et al. [120]. Levels of *CYP1B1* in oral tissue were approximately 2–4 folds higher in smokers than in nonsmokers according to a recent report by Boyle et al. [121]. In addition, Sacks et al. [122] stated that the approximate level of 3–5 *μ*g/mL of tobacco smoke particles would enhance epithelial oral cells. Thereby, regarding to the ability of tobacco smoke particles to induce *CYP1B1* in cultured human cells and hence in smoker oral tissue, there is a good correspondence between the established lower concentration range in the Sacks et al. [122], research and levels in oral tissue in smokers.

### 2.3. *CYP2D6*

Cytochrome P450s consist of the major enzymes required for phase I metabolism of xenobiotics. Cytochrome P450 2D6 (*CYP2D6*) is one of the enzymes that catabolize about 20% of commonly prescribed drugs. Cytochrome P450 2D6 has also a variety of activities among human populations. In fact, the interindividual metabolism rates differ more than 10000 folds [123–125]. Furthermore, the *CYP2D6 *gene is activated by some xenobiotic carcinogens such as nicotine which is the major constituent of tobacco [126]. Several predictive computer models have been published in which the distance between a basic nitrogen atom and the site of oxidation in the substrates determines whether a compound is metabolized by *CYP2D6* or not [127]. The *CYP2D6* gene is localized on chromosome 22q13.1 [128]. The variant *CYP2D6* alleles can be classified into categories, which cause catalytic activity abolish, decrease, to stay normal, increase, or to be qualitatively altered.

Some of the known allelic variants of *CYP2D6 *are not functional or have a reduced catalytic activity (http://www.imm.ki.se/cypalleles/). CYP2D6*4 (G1934A) is the most common poor metabolizer (PM) in Caucasians; however, its frequency is very low in Asians [129, 130]. CYP2D6*3 (2549delA), CYP2D6*5, and CYP2D6*6 (1707delT) are also frequent PMs in Caucasians. Yet, they were described in a less frequency in the Asian population [129–131]. CYP2D6*10 allele (C100T at exon 1), related to a reduced catalytic activity, was found in 50% of the Asian populations and in 2% among Caucasians [129, 130, 132].

The role of the *CYP2D6 *gene as a risk factor for tobacco-related cancers has been extensively studied since early reports suggested an association between the high-metabolizing *CYP2D6 *phenotype and HNC risk in smokers [28, 133, 134]. However, no association between *CYP2D6* genotype and smoking dose has been observed in terms of risk for UADT cancer in another study [29, 76]. Recently, Yadav et al. [135] found a difference in the risk of developing HNSCC depending on the genotype. In fact, patients with CYP2D6*4 allele present an increased risk, while those with CYP2D6*10 allele have no change or even a small decrease in risk in the Indian patients when comparison is done between consumers of tobacco or alcohol and nonconsumers. Thus, *CYP2D6* genotypes are not the only genetic factors that interact with environment in determining the susceptibility to HNSCC. Furthermore, it was shown that patients with poor metabolizer genotypes of *CYP2D6* did not respond to the treatment. The fact that the majority of patients present either CYP2D6*4 or CYP2D6*10 genotypes indicates that individuals with PM genotypes of *CYP2D6* are more prone to develop HNSCC [135]. In addition, it was reported that *CYP2D6* ultrarapid metabolizer patients from Spain and Germany have an increased risk to develop HNSCC [75, 136]. Nevertheless, patients with laryngeal SCC and breast cancer have an increased frequency of PM genotypes [56, 137]. These observations are consistent with previously reported results [138, 139]. However, Kato et al. [140] have reported that patients carrying inactivating alleles of the *CYP2D6* gene have reduced levels of DNA nitrosamine. Caporaso et al. [141] have demonstrated that *CYP2D6* is not involved in nicotine dependency, and hence this gene is not likely to have a major effect on tobacco smoking.

### 2.4. *CYP2E1*

The *CYP2E1* human gene is located on chromosome 10 (10q24.3-qter), contains 9 exons, and encompasses several polymorphisms. Some of them have an effect on the protein expression [142]. The *CYP2E1* enzyme is responsible for the metabolism of alcohol and some tobacco carcinogens such as low-molecular weight nitrosamines [24, 143, 144]. *CYP2E1 *enzyme activity is needed during the metabolic activation of many carcinogens such as nitrosamines. *CYP2E1 *is expressed in oral epithelial cell lines cultures, in human oral mucosa, and in tongues of rats [145, 146]. Two linked polymorphisms (CYP2E1*5B) have been described in the *CYP2E1 *gene at nucleotides -1259 and -1019. They are located in the 5′ regulatory region and are detectable by *RsaI* or *PstI *restriction enzyme digestion [*RsaI* is 21053C > T (rs2031920), and *PstI* is 21293G > C (rs3813867), resp.] [142, 147]. According to the presence or absence of these two restriction sites, two alleles have been defined: the common “wild-type” allele (*RasI* ^+^ */PstI* ^−^), known as c1, and the variant allele (*RasI* ^−^ */PstI* ^+^) known as c2. It was suggested that the RasI polymorphism, located in a putative HNF-1 transcription factor-binding site, might play a role in the expression of *CYP2E1 *[142]. In fact, *in vitro* studies have demonstrated that the regulatory region of the c2 homologous allele shows a significant increase in transcriptional acetyltransferase reporter gene if compared with that of the c1 allele [142, 148]. It was also reported that the CYP2E1*6 polymorphism (rs6413432) is suspected to alter transcription of the *CYP2E1 *gene [149].

Over the last two decades, several studies have explored the association of the *CYP2E1* polymorphism with the risk of lung cancer [150], gastric cancer [151, 152], and pancreatic cancer [154]. Recently, several studies on the association between the *CYP2E1 *polymorphism and HNC have also been published, but those studies have yielded contradictory results. Four separate epidemiological studies showed no association between the c2 allelic variant *(RasI* ^−^ */PstI* ^+^) and the risk for UADT cancer in Brazilian [153] or Japanese [55] subjects. Furthermore, Cury et al. [85] and Balaji et al. [154] observed absence of any association with *CYP2E1 PstI* and HNC in Brazilian patients and oral cancer in South Indians. Moreover, Gajecka et al., [56] Tai et al. [71] did not reveal any association between the *CYP2E1 RsaI* polymorphism and the overall risk of larynx cancer in Polish and Chinese patients, respectively. In addition, other studies [53, 55, 134, 155, 156] have not found significant differences in allelic variants in patients with HNSCC, including oral cancer. However, Gajecka et al.  [56] found that *RasI* ^−^ */PstI* ^+^ variant allele was more frequent in controls (2.8%) than in larynx cancer group (1.6%), which may suggest that the mutated allele is rather “protective”. These results are in agreement with the Swedish study which reported that individuals with *RasI* ^−^ */PstI* ^+^ allele may be at lower risk for lung cancer [157]. However, these results are not consistent with previous studies conducted in the Caucasian and Chinese populations, in which the *CYP2E1 RasI* SNP was shown to be associated with increased risk of HNSCC, OSCC, and esophageal cancer [26, 147, 158–160].

Several Brazilian studies have explored the role of *CYP2E1* polymorphisms in the induction of HNC. A later study [68] on Brazilian patients with HNC indicated that the presence of the *RasI* ^−^ */PstI* ^+^ variant allele was associated with an increased risk of suffering, specifically, from oral cancer. Furthermore, in another Brazilian study of HNC [67], it was observed that the CYP2E1*5A/*5B (c1/c2) genotype was more frequent in oral cavity tumors than in tumors from other anatomic sites (*P* = 0.003) and that the CYP2E1*5A/*5A (c1/c1) genotype was more frequently detected in white patients (*P* = 0.0031). A study including 289 Brazilian volunteers showed that the frequencies of the CYP2E1*6 alleles (*DraI*, rs6413432) are similar to those observed in Caucasians and African-Americans, but the frequency of the CYP2E1*5B allele is higher in Brazilians [161]. However, for the Brazilian population, taking into account the small number of nonwhite individuals, conclusions were so limited. Moreover, ancestry informative marker-based reports have concluded that, at an individual level in Brazil, race is a poor predictor of genomic ancestry [162, 163].

The association between *CYP2E1 (RsaI/PstI*) and *CYP2E1 (DraI*) polymorphisms and HNC susceptibility has been widely investigated. However, results were inconsistent. Recently, Lu et al. [164] and Tang et al. [149] have assumed that *CYP2E1 (RsaI/PstI)* polymorphism might be a risk factor for HNC in the Asian population as well as several carcinogenic processes that cpolymorphism might be a risk factor

ould induce carcinogenesis. In contrast to these findings, studies conducted by Liu et al. [165] on a Chinese population have concluded that there is no significant association between *CYP2E1 (RsaI* or *DraI)* polymorphisms and susceptibility to esophageal SCC (OR = 1.67, *P* = 0.11; OR = 1.11, *P* = 0.74, resp.). Therefore, it was suggested that c2 allele and DD genotype represent a risk factor for esophageal SCC. The frequencies of these two mutations in the Chinese population [165] were all higher than those of the Caucasian population, which indicated the ethnic difference in the two polymorphisms of *CYP2E1* [166, 167]. Hence, there might be a reliable efficiency to evaluate genetic susceptibility of *RsaI* and *DraI *polymorphisms for esophageal SCC in a population with high mutant frequencies.

Tobacco and alcohol consumption represent the most important factors for HNC; hence, genes involved in tobacco carcinogen and alcohol metabolism should play a role in the HNC development. An association between the c2 allele and the increased oral cancer risk was previously demonstrated among nonbetel quid chewing males from Taiwan [168]. In addition, in another Taiwanese study [169], the *CYP2E1 (c2/c2)* genotype was found to be associated with an increased NPC risk, an effect most pronounced in non-smokers. Recently, Jia et al. [170] found robust evidence for associations between genetic variants of *CYP2E1* and NPC risk in the Cantonese population. They observed that individuals aged less than 46 years and who had a history of cigarette smoking present OR of specific genotypes ranging from 1.88 to 2.99 corresponding to SNPs rs9418990, rs1536826, rs3827688, and rs8192780 (*P* = 0.0001–0.0140). Furthermore, Liu et al. [146] compared the risk of oral cancer between the Caucasian and African-American patients depending on the genotype. They found that patients with “wild-type” (c1c1) genotype have an increased risk if compared with controls smoking less than 24 pack-years. Nevertheless, this association was absent for patients with CYP2E1 genotypes among heavy smokers. These findings support the hypothesis that impact of genetic factors in cancer risk is more reduced if carcinogen doses are higher [171]. A hospital-based study [172] of CYP2E1*5B and CYP2E1*6 polymorphisms and gene-environment interactions in the risk of UADT cancers among Indians was conducted. Results showed absence of differences between groups for the two polymorphisms if analyzed separately. However, results for CYP2E1*6 polymorphisms showed significant interactions among tobacco smokers (>40 pack-years) and regular tobacco chewers. These results illustrate the interaction between genes and environment and provide an additional genetic risk factor, CYP2E1*6 polymorphisms, for UADT cancers in the Indian population [172].

**Figure 2:**
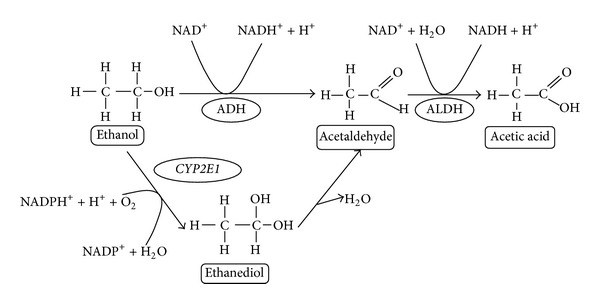
Alcohol metabolism [173].

*CYP2E1* metabolizes ethanol and generates reactive oxygen species, and it has been suggested that it is important for the development of alcoholic liver disease and cancer, including hepatoblastoma and HNC. Acetaldehyde dehydrogenases are a group of *NAD*-dependent enzymes, which catalyze the oxidation of acetaldehyde, being the second enzyme of the alcohol oxidation pathway (Figure 2) [27, 173]. Levels of *CYP2E1* are elevated under a variety of physiologic and pathophysiologic conditions and after acute and chronic alcohol exposure [174, 175]. Interestingly, in a recent Chinese study of Guo et al. [160], the polymorphism of CYP2E1*5B gene (c2/c2 genotype) and alcohol consumption and were found to increase the risk of OSCC (*P* < 0.01, OR = 2.46, and 95% CI = 1.78–4.04). In addition, in the study of Olivieri et al. [67], among alcohol users, the CYP2E1*5B variant allele was more frequently detected in HNC Brazilian patients than in control subjects (*P* < 0.0001, OR = 190.6, and 95% CI = 24.50–1483). Overall, the data suggested that CYP2E1*5B is an independent biomarker of risk in alcohol-related HNC. Recently, Cury et al. [85] confirmed that smoking and alcohol consumption were risk factors for HNC, but the CYP2E1*6 and CYP2E1*5B polymorphisms investigated had no association with the development of HNC in Brazilian patients. Alcohol or tobacco consumptions were also found to interact with variant genotypes of *CYP2E1* in significantly enhancing HNC risk [147]. In addition, it was suggested that CYP2E1*5B polymorphism can be quite important in oral carcinogenesis in Brazilians [53] or can be compensated by other genes involved in the ethanol and other carcinogens metabolism in oral mucosa [53]. Absence of association between CYP2E1*5B polymorphism and lung cancer among patients from Rio de Janeiro has been previously observed [155]; this could be explained by the fact that alcohol is not a lung carcinogen.

The difference in the genetic background between different ethnicities associated to other genetic factors involved in the etiology of HNC might be behind the variability and the inconsistency of these results. It is well established the involvement of some genetic polymorphisms if combined with smoking and alcohol metabolism in the development of HNC. The risk is higher than the tobacco and alcohol consumption is immense. Regarding the genetic component, its effect is depending on the allele combination. Further population genetic studies focusing on metabolizing enzyme polymorphisms should be very helpful in clarifying the individual genetic susceptibility and hence offer the adequate and personalized management of the patient.

## 3. Arylamine *N*-Acetyltransferases (*NATs*)

**Figure 3:**
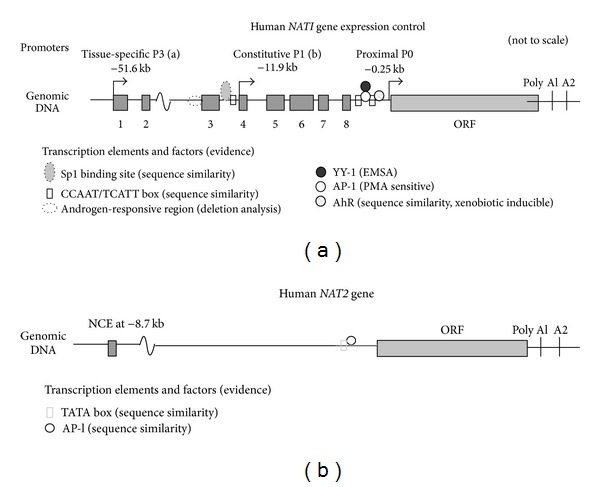
Human *NAT* genes: (a) *NAT1* and (b) *NAT2* [176, 177].

*NAT1* and *NAT2* human isoforms are encoded by two genes with intronic less coding regions. The *NAT* genes are located on chromosome 8p21.3–23.1 and express two highly polymorphic isoenzymes (*NAT1* and *NAT2*) with distinct functional roles. In humans, the products of these two genes appear to have distinct functional roles depending on their substrate, their expression in tissues, and the expression of the different genes during development. Although the two genes are organized in a single open-reading frame, their structure and control vary markedly (Figure 3) [176, 177]. Recent studies on human *NAT1* and *NAT2* genes have identified interactions within the active site cleft that are crucial for substrate recognition [178]. The specific recognition of the substrate is provided by the C-terminal region of the *NAT* proteins [179], mainly by residues around positions 124–129 [180].

*N*-Acetyltransferases are involved in the metabolism of certain carcinogens responsible for tumors in rodents like aromatic and heterocyclic amine carcinogens [181]. Based on genetic engineering, a critical cysteine (amino acid 68) within the catalytic site was created. This catalytic site is implicated in acetyl transfer between the acetyl-CoA cofactor and acceptor substrates [182]. The latter could be aromatic amines and hydrazines (*N*-acetylation) or *N*-hydroxy-aromatic and *N*-heterocyclic amines (*O-*acetylation). The substrate could be activated or deactivated by *NAT1* and/or *NAT2* if *O-*acetylated or *N*-acetylated, respectively [183]. Because these two genes are involved in metabolic activation *via O-*acetylation [184–187], their genetic polymorphisms could modify the cancer susceptibility related to carcinogen exposure. Many *N*-hydroxy heterocyclic amine carcinogens are catalyzed by human *NAT2* than *NAT1* [185, 187]. Their tissue-specific expression is also a determinant factor for a better efficiency.

So far, 36 *NAT2* genetic variants have been identified in human. Among them, NAT2*4 is the most common allele reported to be associated with rapid acetylation [188]. The other alleles are classified into two groups: the rapid alleles that include NAT2*11A, NAT2*12A-C, NAT2*13A, and NAT2*18 and the slow alleles such as NAT2*5, NAT2*6, and NAT2*7. For *NAT1*, the most common alleles are NAT1*3, NAT1*4, NAT1*10, and NAT1*11. NAT1*4 is the most common allele, while NAT1*10 is the putative rapid allele. Subjects having more than one rapid allele were designated by *NAT1* rapid acetylation. For the others, they were classified under *NAT1 *slow acetylation [188].

**Figure 4:**
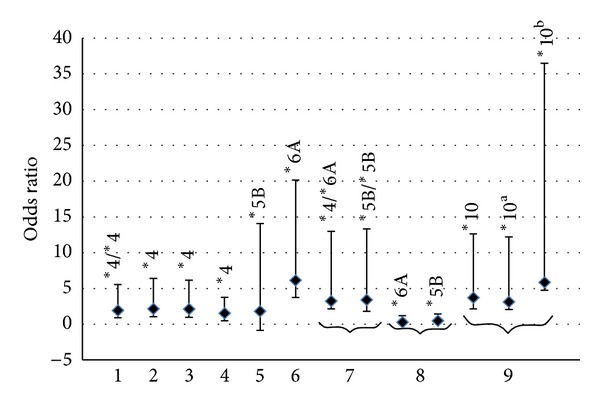
Odds ratios (OR) for HNCs from one *NAT1* and eight *NAT2* studies. Bars indicate 95% confidence intervals (CI), while individual SNPs in each study are labeled for each vertical line, and study numbers are indicated at the bottom (*NAT2,* (1: [53]; 2: [195]; 3: [197]; 4: [198]; 5: [194]; 6: [193]; 7: [56]; 8: [133]; *NAT1*, 9: [199]). ^a^Smokers; ^b^nonsmokers.

As *NAT1* and *NAT2* genes are characterized by allelic heterogeneity, several haplotypes have been established. They were associated with either the rapid or the slow acetylator phenotype [133]. All SNPs of both genes (slow and rapid alleles) have been associated with an increased risk of cancer. This association could be explained by their ability to detoxify aromatic amine carcinogens from one hand and to produce higher levels of reactive metabolites from another hand [189]. In 1987, Drozdz et al. [190] had established an association between the slow acetylator phenotype and the increased risk for laryngeal cancer. So far, little is known about the role of the *NAT* gene SNPs and their association with HNC (Figure 4).

Some studies have reported that alteration of NAT enzyme activity might be of risk for UADT cancer. It was previously shown that patients with *NAT2* slow acetylator genotypes (homozygous for NAT*5, NAT*6, and NAT2*7 alleles) are significantly (*P* < 0.002) more prone to develop UADT cancer (0.37) as compared with controls (0.22) [133]. In a recent study on the Turkish population, the slow acetylator NAT2*7 allele was correlated to a reduced UADT cancer risk [191] as well as larynx cancer [192], thus suggesting a protective role of NAT2*7 genotype in HNC. NAT2*5 and NAT2*6 alleles seem to be associated with cancer risk [191]. Studies focusing on *NAT2* haplotypes have shown an association between NAT2*4 and HNC [191]. These results support the hypothesis of the possible involvement of NAT2*4 combinations (NAT2*4/*6A) in larynx cancer predisposition (OR = 3.24; *P* = 0.045) [56]. In a Tunisian study, Bendjemana et al. [193] observed that genotypic frequencies of NAT2*6/NAT2*6 were significantly higher in the group of nasopharyngeal carcinoma patients (OR = 6.14; 95% IC = 2.4–14.0). Furthermore, in another Tunisian study [194], a significant difference was found between HNC patients and controls for T341C mutation (NAT2*5, rs1801280) in *NAT2* gene (OR = 1.82; *P* = 0.04). This finding is in accordance with the reported association between squamous cell carcinoma and T341C mutation [133]. This is probably due to the great reduction in acetyltransferase 2 catalytic activity in relation with the T341C mutation (NAT2*5) in *NAT2* gene [189]. However, no significant difference was found between HNC Tunisian patients and controls for G590A (NAT2*6) mutation in *NAT2* gene [194]. In addition, no association between the *NAT2 *genotype and NPC was found in the Taiwanese population [77].

An association was found between the homozygous NAT2*4 allele and the increased oral cancer risk in a Brazilian population (OR = 1.95; *P* = 0.032) [53]. Likewise, many studies have reported an association between rapid acetylator phenotype and the increased risk of oral and laryngeal cancer in Caucasians [195, 196]. At the biological level, this could be explained by the fact that *O-*acetylation of nitrosamines by *NAT2 *could be more important as a negative metabolic pathway leading to oral carcinogenesis; therefore, slow acetylators would be protected. Chatzimichalis et al. [197] established the distribution of genotypes and showed that it consisted of 55.68% of rapid acetylators and 44.32% of slow acetylators in laryngeal SCC patients, while it was of 36.27% of rapid acetylators and 63.72% of slow acetylators in controls. This study [197] concluded that rapid acetylator genotypes are significantly associated to laryngeal SCC in the Greek population (OR = 2.207; *P* = 0.0087). Furthermore, Buch et al. [198] found that fast acetylators (NAT2*4) are more frequent in oral cancer patients (53.7%) than in controls (43.9%; OR = 1.55; 95%CI = 1.08–2.20; *P* = 0.03).

Several studies have explored the role of *NAT1 *polymorphisms in the incidence of HNC. The first study [199] was conducted to test the oral cancer risk associated with polymorphism in the *NAT1* gene. This study is still until now the only one that suggested a significant increased risk (OR = 3.72; *P* < 0.01) associated with the NAT1*10 allele in the Japanese population [199]. However, the other studies have suggested negative findings [191, 195, 200, 200].

Gene-gene interaction testing has shown several cancer-*NAT2* associations. The strongest one was observed among persons without a *CYP1A1* variant (*2C or *4) allele (OR = 1.77, 95% CI = 1.20–2.60, and *P* = 0.03) [198]. These results implicate fast *NAT2 *acetylation as a risk factor for oral cancer in the American population (USA) [198]. Moreover, Demokan et al. [191] and McKay et al. [201] found that the association with *NAT1* and *NAT2 *gene combinations may influence the risk of developing HNC. A significant association was observed between the fast acetylator NAT2*4/NAT1*10 diplotype and risk of HNC [191]. Moreover, the association with NAT1*11/NAT2*6A haplotypes was correlated to the risk of UADT cancer (OR = 1.54; *P* = 0.03) [201].

*NAT* gene presents a crucial role in the detoxification and activation reactions of numerous xenobiotics originating not only from tobacco-derived aromatic and heterocyclic amine carcinogens but also from drug metabolism. Its function is undergone through *N*- and *O-*acetylation pathways [200, 202] *via* a ping-pong bi-bi mechanism. The initial step consists on acetylation of Cys68 by an acetyl-coenzyme A along with the release of the cofactor product coenzyme A. Secondly, the substrate is linked to the acetylated enzyme. Finally, the acetylated product is released [203]. Since chemical compounds present in tobacco are inactivated by phase II enzymes, it has been proposed that HNC risk could be modified by *NAT* genotypes. HNCs are strongly associated with smoking, and a few studies have explored the role of *NAT1* polymorphisms in the risk of developing HNC in smokers [199, 204]. However, overall findings are inconsistent, and associations if present are weak and indicate either a decreased risk in carriers of the variant *NAT1* [201], an increased risk [205], or a lack of association [191, 195, 200, 202].

The role of *NAT1* and *NAT2* acetylator polymorphisms in cancer risk from aromatic and heterocyclic amine carcinogens will become clearer with more precise determinations of both exposures and genotypes. Further studies of the haplotype combinations in different populations and with larger cohorts are warranted to determine the range of risks associated with the effect of genetic variation of the NAT genes with regard to HNC.

## 4. Conclusion

The present paper reviews studies that assessed association between genetic polymorphisms of genes encoding carcinogen-metabolizing enzymes and showed their possible involvement by significantly increasing the predisposition for HNC. This risk relies on many factors such as the level of carcinogen exposure (e.g., tobacco smoke), the ethnicity and/or racial groups, and so forth. Various polymorphisms in these genes are summarized in Table 2. Many of the discussed studies described HNC risk for a mixed racial and/or ethnic cohort. As shown previously, cancer susceptibility is different according to the genotype in a given racial group. Thus, even if cases and controls are race-matched, erroneous association might be taken into consideration if different racial and/or ethnic groups are mixed. In addition, differences in genetic backgrounds for metabolic genotypes between races and even between ethnic groups whether located in the same region or not should also be taken into consideration before an association study is performed. Furthermore, metabolizing enzyme expression could widely vary at diverse sites within the head and neck.

It is well known that there is a real logistical difficulty that consists in combating at least one of the potential biases listed above. However, careful attention should be given to all elements before conducting an association study in order to ensure accurate and significant results. If well designated, these studies would clarify the impact of xenobiotic-metabolizing enzymes in HNC development and help determine the value of potentially “high-risk” genotypes in HNC prevention strategies.

## Figures and Tables

**Table 1:** Polymorphic CYPsP450 and NATs and metabolism of carcinogens in the HNC.

Gene	Substrate	Reference	Functional effect	Polymorphism	rs number	Reference
*CYP1A1 *	TC^a^ (e.g., benzo[a]pyrene dimethylbenz[a]anthracene), 6-nitrochrysene,	[33]	Phase I oxidative and reductive	CYP1A1*2A (m1): T→C substitution at nucleotide 6235 in the 3′ noncoding region	—	[35]
				CYP1A1*2B (m2): A→G Ile462Val	rs1048943	
				CYP1A1*3 (m3): T→C substitution at nucleotide 5996 in the 3′ noncoding region	—	
				CYP1A1*4 (m4): C→A Thr461Asn	rs1799814	

*CYP1B1 *	TC^a^ (e.g., benzo[a]pyrene)	[92]	Phase I oxidative and reductive	CYP1B1*3: Val432Leu	rs1056836	[101]
				CYP1B1*2: Arg48Ser	rs1056827	
				CYP1B1*2: Ala119Ser	rs10012	
				CYP1B1*4: Asn453Ser	rs1800440	

*CYP2D6 *	TC^a^ (e.g., nicotine and nitrosamines)	[126]	Phase I oxidative and reductive	CYP2D6*3: (single-base deletion at exon 5, 2549delA)	rs35742686	[129]
				CYP2D6*4: (G1934A)	rs3892097	
				CYP2D6*5: (leading to gene deletion)	—	
				CYP2D6*6: (single-base deletion at exon 3, 1707delT)	rs5030655	

*CYP2E1 *	TC^a^ (e.g., nitrosamines) Ethanol	[24, 143, 144]	Phase I oxidative and reductive	CYP2E1*5B (c2) (PstI restriction, position: −1019)	—	[142]
				CYP2E1*5A (c1) (RsaI restriction, position: −1259)	—	
				CYP2E1*6 (allele D) (DraI restriction, intron 6)	rs6413432	[149]

*NAT1/NAT2 *	Arylamines and heterocyclic aromatic amines	[181, 184, 186, 187]	Phase II biotransformation	NAT1*10: T1088A	rs1057126	[188]
				NAT1*10: C1095A	rs15561	
				NAT2*5B: Ile114 Thr, NAT2*5B: Lys268Arg	rs1801280 rs1208	
				NAT2*6A: Arg197Gln	rs1799930	

^a^Tobacco carcinogens; —: undefined.

**Table 2:** Summary of studies on CYPs450 and *NATs* genes status in HNC.

Gene	Population	*N* (case/control)	SNP (allele or genotype)	OR	95% CI	*P* value	References
*CYP1A1 *	Japanese	100/100	*2A/*2A	3.6	1.4–9.5	<0.05	[69]
	Indian	—	*1A/*2A	1.76	1.19–2.60	—	[66]
			*2A/*2A	2.83	1.43–5.61	—	
	Indian	458/729	m2/m2	3.2	1.10–10.28	0.05	[64]
	Korean	72/221	m2/m2	3.8	1.9–7.7	0.023	[63]
	Brazilian	153/145	*1A/*2A	—	—	0.003	[67]
	Japanese	142/142	m2/m2	2.3	1.1–4.7	<0.05	[54]
	Indian	408/220	MspI^a+^	6.43	3.69–11.21	<0.05	[65]
			MspI^c+^	10.24	5.95–17.60	<0.05	
	Brazilian	103/102	MspI^+^	2.4	1.13–5.10	—	[68]
	Indian	203/201	T3801C	4.89	3.15–7.32	<0.01	[89]
	Japanese	145/164	*2A/*2A	4.1	1.1–15	0.038	[55]
	Chinese	278/278	3798 CC	2.39	1.11–5.16	0.027	[71]
	Indian	205/245	m1/m1	8.12	3.27–21.30	0.000002	[79]
			w1/m1	1.96	1.14–3.38	0.0092	
			m2/m2	6.31	2.24–18.69	0.000015	
	Polish	289/316	*4/*4	1.70	0.99–2.88	0.049	[56]
	Caucasian (meta-analysis)	—		1.29	1.05–1.65	—	[70]
	22 studies (meta-analysis)	4168/4638	MspI^+^	—	1.15–1.57	<0.001	[59]
			MspI^a^	2.98	1.69–5.26	<0.001	

*CYP1B1 *	Indian	150/150	*2 (wt/mt)	2.36	1.27–4.38	0.04	[21]
			*2 (mt/mt)	3.34	1.20–9.36	0.03	
			*2 (wt/mt)^a^	2.37	1.62–4.85	<0.05	
			*2 (wt/mt or mt/mt)^a^	4.47	2.07–9.60		
			*2 (wt/mt or mt/mt)^c^	8.81	2.60–29.87	<0.05	
			*3 (wt/mt or mt/mt)^c^	2.74	1.12–6.70	<0.05	
	German	195/177	*3^a, f^	4.53	2.62–7.98	<0.001	[26]

*CYP2D6 *	Indian	350/350	*4 (mt/mt)	2.32	1.14–4.34	<0.001	[135]
			*10 (wt/mt)	2.06	1.48–2.87	<0.001	
			*10 (mt/mt)	1.85	1.19–2.89	<0.001	
	Polish	289/316	*4/*4 (1934GG)	2.36	1.03–5.39	0.045	[56]

*CYP2E1 *	Brazilian	153/145	*5B	190.6	24.50–1483	<0.0001	[67]
	Chinese	320/320	*5B	2.46	1.78–4.04	<0.01	[160]
	Indian	—	*5B	3.44	1.45–8.14	—	[147]
			*6	1.76	1.45–2.41	—	
	German	312/299	−71 G>T	0.49	0.25–0.98	0.04	[159]
	Chinese	755/755	rs9418990^d^	2.95	1.68–5.17	0.0002	[170]
			rs8192780^d^	2.99	1.72–5.21	0.0001	
			rs1536826^d^	2.94	1.69–5.13	0.0001	
			rs3827688^d^	1.88	1.13–3.13	0.0140	
	CaucasianAfrican-American	113/226	c1/c1^e^	—	—	0.033	[146]
		58/173					
	24 studies (meta-analysis)	12,562/—	*5B				[164]
			c2 allele	1.11	1.00–1.22	0.04	
			c2/c2	1.57	1.14–2.15	0.006	
	17 studies (meta-analysis)	1,663/2,603	c1/c2	0.64	0.50–0.81	<0.001	[158]
	24 studies (meta-analysis)	12,562 (all)	c2/c2	1.57	1.14–2.15	0.006	[206]
	21 studies (meta-analysis)	4,951/6,071	*5B	1.96	1.33–2.90	<0.05	[93]
			*6	1.56	1.06–2.27	<0.05	
	Asian (meta-analysis)	4,951/6,071	*5B	2.04	1.32–3.15	<0.05	[93]
			*6	2.04	1.27–3.29	<0.05	

*NAT1 *	Japanese	62/122	*10	3.72	1.56–8.90	<0.01	[199]
			*10^a^	3.14	1.09–9.07	0.017	
			*10^b^	5.88	1.13–30.6	0.022	

*NAT2 *	Brazilian	231/212	*4/*4	1.95	1.05–3.60	0.035	[53]
	German	255/510	*4	2.18	1.13–4.22	0.018	[195]
	Greek	88/102	*4	2.20	1.23–3.95	0.0087	[197]
	American (USA)	203/416	*4	1.55	1.08–2.20	0.03	[198]
	Tunisian	64/160	*5B	1.82	2.68–12.26	0.04	[194]
	Tunisian	45/100	*6A	6.14	2.4–14	<0.05	[193]
	Polish	289/316	*4/*6A	3.24	1.1–9.75	0.045	[56]
			*5B/*5B	3.41	1.6–9.9	0.043	
	Spanish	75/200	*6A	0.30	0.10–0.89	<0.042	[133]
			*5B	0.48	0.25–0.93	<0.039	
		145/164	—	2	—	0.039	[55]

^a^Smokers; ^b^nonsmokers; ^c^chewers; ^d^smokers <46 years; ^e^subjects smoked <24 pack-years;^ f^calculation for wt/wt genotype versus wt/mt and mt/mt genotypes.

—: undefined; +: the genotype/allele undefined.

## Abbreviations

CYP450: Cytochrome P450
HNSCC: Head and neck squamous cell carcinoma
NPC: Nasopharyngeal cancer
OR: Odds ratios
OSCC: Oral squamous cell carcinoma
NAT: Arylamine *N*-acetyltransferase
PM: Poor metabolizer
SCC: Squamous cell carcinoma
SNP: Single-nucleotide polymorphism
TSNAs: Tobacco-specific nitrosamines
UADT: Upper aerodigestive tract.
